# Supplementary material for: Identification of photoperiod-induced specific miRNAs in the adrenal glands of Sunite sheep (Ovis aries)
Source: Front Vet Sci. 2022 Jul 22;9:888207. doi: 10.3389/fvets.2022.888207 (PMC9354845; doi:10.3389/fvets.2022.888207)
Supplement: Supplementary file 1 [file Data_Sheet_1.ZIP › Supplementary file/miRDeep2 file.zip!/miRDeep2 file/Know_Structure/SPLP42A3/oar-mir-379.pdf]

miRBase precursor : oar-mir-379
Total read count : 49537
oar-miR-379-5p read count : 47768
oar-miR-379-3p read count : 1745
remaining reads : 24

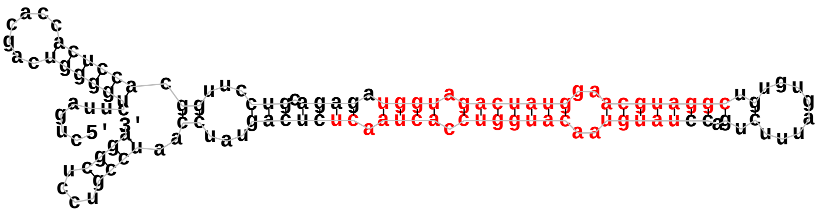

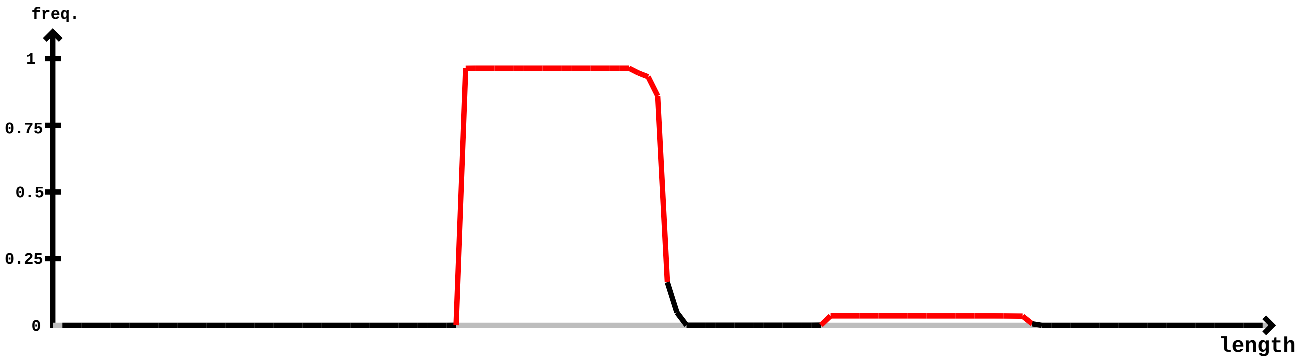


```
                                                                               oar-miR-379-3p
                                          oar-miR-379-5p
5'- cugauucuggggucagcaccacuccacgguuccugcagagaugguagacuauggaacguaggcugugugauuucugaccuauguaacaugguccacuaacucucaguauccaauccguccucgga   -3'    exp
    .......((((((.......)))))).((...(((.((((.((((.(((((((..((((((((.(........).).)))))))..))))))).)))).)))))))...))..((((....))))    reads      mm
    ........................................augguagacuauggaacgua................................................................    1          0
    ........................................augguagacuauggaacguag...............................................................    3          0
    ........................................augguagacuauggaacguagg..............................................................    10         0
    .........................................ugguagacuauggaacg...................................................................    1          0
    .........................................ugguagacuauggaacgu..................................................................    902        0
    .........................................ugguagacuauggaacgua.................................................................    694        0
    .........................................ugguagacuauggaacguag................................................................    3556       0
    .........................................ugguagacuauggaacguagg...............................................................    34608      0
    .........................................ugguagacuauggaacguaggc..............................................................    5629       0
    .........................................ugguagacuauggaacguaggcu.............................................................    2347       0
    .........................................ugguagacuauggaacguaggcugu...........................................................    2          0
    ..........................................gguagacuauggaacguagg...............................................................    10         0
    ..........................................gguagacuauggaacguaggc..............................................................    3          0
    ..........................................gguagacuauggaacguaggcu.............................................................    1          0
    ...........................................guagacuauggaacguagg...............................................................    1          0
    ..........................................................uaggcugugugauuucugaccu.............................................    1          0
    ..............................................................cuAugugauuucugacc..............................................    1          1
    ..............................................................cugugugauuucugacU..............................................    8          1
    ..............................................................cugugugauuucugacc..............................................    12         0
    ...............................................................ugugugauuucugaccuauguaacau....................................    2          0
    ..............................................................................cuauguaacaugguccacu............................    1          0
    ..............................................................................cuauguaacaugguccacuaac.........................    21         0
    ..............................................................................cuauguaacaugguccacuaacu........................    1          0
    ...............................................................................uauguaacaugguccacC............................    1          1
    ...............................................................................uauguaacaugguccacu............................    4          0
    ...............................................................................uauguaacaugguccacua...........................    13         0
    ...............................................................................uauguaacaugguccacuaa..........................    16         0
    ...............................................................................uauguaacaugguccacuaac.........................    1408       0
    ...............................................................................uauguaacaugguccacuaacu........................    278        0
    ...............................................................................uauguaacaugguccacuaacuc.......................    1          0
    ................................................................................auguaacaugguccacuaac.........................    1          0
```
